# Prostacyclin Synthase: Upregulation during Renal Development and in Glomerular Disease as well as Its Constitutive Expression in Cultured Human Mesangial Cells

**DOI:** 10.1155/2015/654151

**Published:** 2015-01-18

**Authors:** Thomas Klein, Günther Klaus, Martin Kömhoff

**Affiliations:** ^1^Department of Pediatrics, University of Marburg, 35043 Marburg, Germany; ^2^Boehringer Ingelheim, Pharma Division Research, 88397 Biberach, Germany

## Abstract

Prostacyclin (PGI_2_) plays a critical role in nephrogenesis and renal physiology. However, our understanding of how prostacyclin release in the kidney is regulated remains poorly defined. We studied expression of prostacyclin synthase (PGIS) in developing and adult human kidneys, and also in selected pediatric renal diseases. We also examined PGI_2_ formation in human mesangial cells* in vitro*. We observed abundant expression of PGIS in the nephrogenic cortex in humans and* in situ* hybridization revealed an identical pattern in mice. In the normal adult kidney, PGIS-immunoreactive protein and mRNA appear to localize to mesangial fields and endothelial and smooth muscle cells of arteries and peritubular capillaries. In kidney biopsies taken from pediatric patients, enhanced expression of PGIS-immunoreactive protein was noted mainly in endothelial cells of patients with IgA-nephropathy. Cultured human mesangial cells produce primarily PGI_2_ and prostaglandin E_2_, followed by prostaglandin F_2_ *α* Cytokine stimulation increased PGI_2_ formation 24-fold. Under these conditions expression of PGIS mRNA and protein remained unaltered whereas mRNA for cyclooxygenase-2 was markedly induced. In contrast to its constitutive expression* in vitro*, renal expression of prostacyclin-synthase appears to be regulated both during development and in glomerular disease. Further research is needed to identify the factors involved in regulation of PGIS-expression.

## 1. Introduction

Prostacyclin synthase (PGIS) is an atypical cytochrome p450 enzyme [1], which generates prostacyclin (PGI_2_) from prostaglandin H_2_ (PGH_2_), provided by cyclooxygenase-1 (COX1) or COX2. Prostaglandin-I synthase is expressed constitutively, consistent with its TATA-less and GC-rich promoter [2]. Modulation of constitutive expression with strong upregulation has been observed in uterine development [3]. However,* in vitro* studies have failed to identify specific factors that induce consistently PGIS-protein expression. Prostacyclin, which has a half-life of 30 sec* in vivo*, activates adenylate cyclase through interaction with its G-protein-coupled receptor, dubbed IP [4]. In contrast to certain synthetic and stable prostacyclin analogues, known to activate the nuclear receptor peroxisome proliferator-activated receptor, *β*/*δ* (PPAR-*β*/*δ*) [5], there remains controversy over whether this is also true for endogenous prostacyclin [6, 7].

Development of severe glomerular, vascular, and interstitial abnormalities in PGIS-knockout-mice only serves to confirm the critical role that prostacyclin plays in normal renal development [8]. Such defects are not observed in the IP-knockout-mice [9], fitting with the notion of a second PGI_2_-receptor, possibly PPAR-*β*/*δ*. Numerous glomerular actions of PGI_2_ have been reported, including effects on local hemodynamics, renin secretion, cell proliferation, and matrix turnover [4]. The potential beneficial effects of various synthetic ligands on slowing progression of renal disease have been observed in experimental animal models as well as in humans [4].

Despite the fact that prostacyclin has been shown to play several roles in the kidney, the pattern of PGIS expression in humans during renal development and in glomerular disease is virtually unknown. There are also few studies describing the* in vitro* expression of prostacyclin synthase. In human endothelial cells, biosynthesis of prostacyclin is controlled primarily through the induction of cyclooxygenase by growth factors or mitogens whereas expression of PGIS remains unchanged [10]. Others have shown that bovine endothelial cells translate tumour necrosis factor (TNF) binding into an increase of COX2 enzyme expression and subsequently into the induction of prostacyclin synthase mRNA [11]. Finally, incubation of human endothelial cells with acidic fibroblast growth factor in the presence of heparin resulted in a marked diminution in PGI_2_ synthesis caused by a decrease in expression of both prostacyclin synthase and cyclooxygenase protein [12].

Most studies that have reported on prostacyclin synthesis in the scientific literature have focused on expression and regulation of cyclooxygenases, the enzymes that provide PGH_2_ and thus cyclooxygenases are considered to be the rate-limiting step during prostanoid synthesis. Two isoforms of cyclooxygenase have been identified: a constitutive COX1 that is thought to be involved in housekeeping functions of prostaglandins and an inducible COX2 that is believed to be involved in the synthesis of high amounts of prostaglandins under pathological conditions [13].

The present series of studies were conducted under various different circumstances in order to gain further insight into the regulation of prostacyclin synthase. We analyzed the mRNA and protein expression of prostacyclin synthase in normal “developing” and adult human kidneys, as well as in glomerular disease. We also investigated prostacyclin synthesis and synthase in human mesangial cells* in vitro*.

## 2. Material and Methods

The current work was conducted with permission of our institution's local medical ethics committee. During the course of normal clinical practice, renal tissue was obtained from the unaffected poles of kidneys surgically removed as part of treatment of renal carcinoma (*n* = 5 patients). Human fetal tissue was collected from medical abortions [14]. Unused paraffin-embedded renal biopsy tissue samples remaining after sectioning were also identified for study. In total 21 patients, 12 patients with IgA-nephropathy, seven patients undergoing routine renal biopsy, one patient with chronic renal transplant rejection, and one patient with focal segmental glomerulosclerosis provided consent for their biopsy samples to undergo the study.

### 2.1. Immunohistochemistry of Paraffin-Embedded Adult Human Tissue and Renal Biopsies

Specificity of all primary antibodies has been rigorously tested and confirmed by colocalization studies of the respective mRNA using radioactive* in situ* hybridization [15, 16]. Sections were incubated with rabbit anti-PGIS polyclonal antibodies, as described previously [16]. Primary anti-cyclooxygenase antibodies were obtained from Santa Cruz (Santa Cruz, CA: COX1, C20: sc#1752, and COX2: C-20, sc#1745).

### 2.2. Immunohistochemistry of Human Fetal Tissue

The characterization of the monoclonal antibodies for prostacyclin synthase has been published previously [17]. In brief, approximately 5 *μ*m tissue sections were sliced from snap-frozen samples, thaw-mounted on poly-L-lysine-coated glass slides, air-dried, and fixed in acetone for 10 min at 4°C. The presence of primary antibodies was confirmed with the alkaline phosphatase antialkaline phosphatase method using rabbit anti-mouse or mouse anti-rabbit antibodies.

### 2.3. Generation of 35S-Labelled Riboprobes and* In Situ* Hybridization

Antisense and sense probes for human prostacyclin mRNA were prepared as follows. A polymerase chain reaction (PCR) fragment (420 base pairs) generated the amplification of mesangial mRNA with the primer pair depicted below that was ligated and cloned in a pCR 2.1 plasmid (Invitrogen, USA). The following primers were used to generate a murine-PGIS riboprobe: forward-GGCTGGCTGGGTTGAGAATC and reverse-GACCGTGCGAAGGTTGGTAT. Cloned cDNA fragments were sequenced according to the dideoxy method to confirm the identity and orientation of the inserts.* In situ* hybridization was performed as described previously [16]. After development in Kodak D-19, slides were counterstained with hematoxylin. Photomicrographs were taken with a Zeiss Axioskop microscope using bright field optics.

### 2.4. Human Mesangial Cells

Normal human kidney tissue was obtained from tumor nephrectomy surgery. Glomeruli were obtained from different donors by passage through serially graded sieves and incubated in growth medium which consisted of RPMI-1640 supplemented with insulin (5 *μ*g/mL), transferrin (5 *μ*g/mL), sodium selenite (5 *μ*g/mL), L-glutamine (1%), penicillin (100 U/mL), and streptomycin (100 *μ*g/mL) containing 10% fetal calf serum and HEPES (20 mM). Cellular outgrowths of mesangial appearance were subcultivated and characterized as follows: (a) morphological criteria with elongated appearance; (b) staining with antivimentin; (c) staining with smooth muscle cell actin; and (d) absence of staining with anti(factor VIII) and antidesmin. Cells were grown to confluence and growth was arrested by low serum conditions (0.5% fetal calf serum) in the absence of supplements for 24–36 h. All experiments were performed using cells between the third and sixth passages.

### 2.5. Cell Culture

Cell culture media were obtained from PAA (Coelbe, FRG). Recombinant human (rh) interleukin (IL) types 1*β* IΛ-1*β* and rh-TNF*α* were purchased from Endogene (MA, USA). A commercial enhanced chemiluminescence detection kit with nitrocellulose membranes (Hybond-C) was obtained from Amersham (Braunschweig, FRG). Diclofenac and bovine serum albumin were from Sigma (Deisenhofen, FRG). Primers were synthesized by MWG-Biotech (Ebersberg, FRG); AmpliTaq polymerase was obtained from Perkin-Elmer (Weiterstadt, FRG) and SuperScript reverse transcriptase from GIBCO (Eggenstein, FRG). Insulin, transferrin supplements, and recombinant human interferon (IFN) type-*γ* rh-IFN-*γ* were from Boehringer (Mannheim, FRG). Primary antibodies to characterize cellular cultures were obtained from Dako (CA, USA); secondary antibodies were from Dako and Dianova (Hamburg, FRG).

### 2.6. Western Blot and mRNA Analysis

Analysis of prostacyclin synthase expression was performed on human mesangial cells. In brief, lysates (80 *μ*g) of cells stimulated for 20 h with rh-TNF*α* (100 ng/mL), IFN-*γ* (350 U/mL), IL-1*β* (1 nM), or control were solubilized in phosphate buffered saline containing 1% Triton X-100. Equal amounts of protein were separated by 10% SDS-PAGE. Immunoblot analysis was performed with different purified polyclonal antibodies against PGIS as described previously [17]. Total RNA was isolated from mesangial cells using the guanidinium thiocyanate method with acidic phenol. Reverse transcription and PCR were performed as described recently for COX1, COX2, *β*-actin, and thromboxane synthase. Accordingly, for prostacyclin synthase, 1 *μ*g of total RNA was used for target-specific reverse transcription with reverse transcriptase and the primer (5′-ATGCGGTAGCGGACGACGGGCACG-3′). Polymerase chain reaction amplification was performed using the cDNA with the antisense (5′-ctgcatcagaccgaagccc tacctg-3′) and sense primer (5′-TGCTGAGTGAGAGCCTCAGGCTTA-3′). Reactions were cycled 30 times (30 sec at 94°C, 30 sec at 56°C, and 30 sec at 72°C following a 5 min denaturing step at 95°). Amplification products were analyzed by 1.5% agarose gel electrophoresis and ethidium bromide staining. The identity of the fragments was evaluated by their molecular mass and dideoxy sequencing. Samples were assayed at various dilutions to ensure proportionality in the yield of PCR products.

Determination of prostanoids by gas chromatography is as follows. Prostanoids in the supernatant of mesangial cells were measured by gas chromatography coupled to dual mass spectrography (GC/MS/MS) as described previously [18].

## 3. Results

### 3.1. Intrarenal Localization of Prostacyclin Synthase

**Figure 1:**
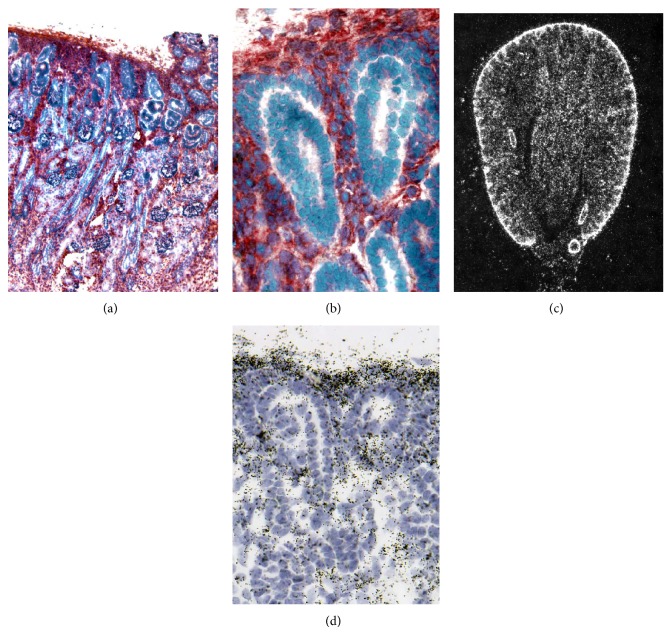
Mesenchymal expression of prostacyclin synthase in the developing human (24 weeks of gestation) and murine (postnatal day 0) kidney. (a) shows a low power view of a developing human kidney. (b) High power reveals absence of PGIS in epithelial structures. (c) Low power shows high subcapsular expression of PGIS-mRNA. (d) confirms the absence of labeling over epithelial structures.

*Nephrogenesis*. In the developing human kidney, intense expression of PGIS was observed in mesenchymal cells of the nephrogenic cortex (Figure 1(a)). Epithelial structures corresponding to various stages of renal development and tubular structures were not labeled (Figure 1(b)). An identical pattern was observed when analyzing prostacyclin synthase mRNA expression in the mouse (Figure 1(c), low power view).* In situ* hybridization revealed strong cortical labeling in kidneys on postnatal day 0. Within the exception of vascular structures, medullary areas showed significantly less labeling. High power views confirmed labeling over interstitial cells and sparing of epithelial structures (Figure 1(d), high power view).

**Figure 2:**
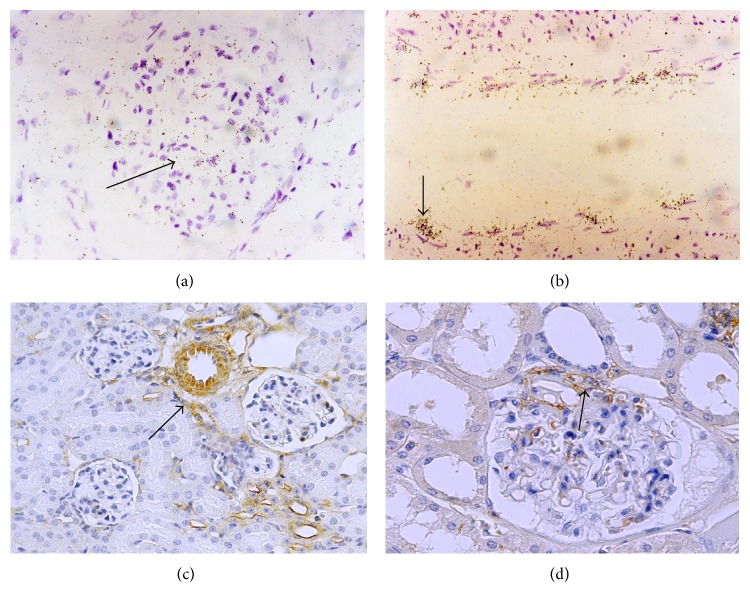
PGIS mRNA localizes to mesangial fields ((a), arrow) and endothelial cells of an interlobar artery ((b), arrow). Expression of PGIS protein and mRNA in normal adult kidney. (c) Low power view shows expression of PGIS in endothelial and smooth muscle cells of an arcuate artery (arrow). (d) shows expression of PGIS in cells of the juxtaglomerular apparatus (arrow), adjacent to the macula densa.

*Normal Adult Kidney*. Radioactive* in situ* hybridization revealed specific signals for prostacyclin synthase in the mesangial region of glomeruli (Figure 2(a)) and over arterial endothelial cells (Figure 2(b)). As expected from biological and pharmacological studies, PGIS immunoreactivity was detected in endothelial cells of blood vessels (Figure 2(c)) of normal kidney as well as in the smooth muscle cells of arteries. Staining of PGIS in the glomerulus was comparatively low in normal human tissue. Note PGIS immunoreactivity in cells of the juxtaglomerular apparatus adjacent to cells of the macula densa (Figure 2(d)).

**Figure 3:**
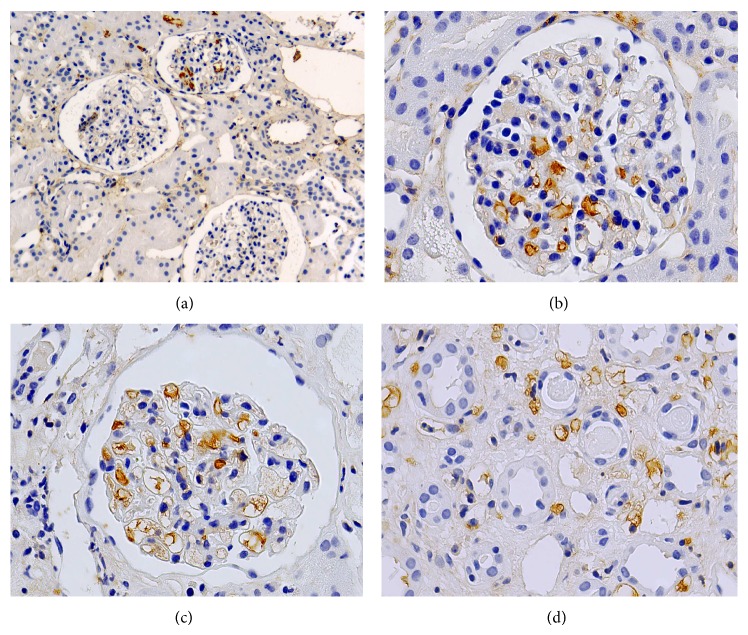
Expression of prostacyclin synthase in various renal diseases. (a) IgA-nephropathy, (b) focal segmental glomerulosclerosis. Glomerular (c) and medullary (d) expression of PGIS in endothelial cells and peritubular capillaries in a transplanted kidney with chronic rejection.

*Pediatric Renal Disease*. Glomerular expression of PGIS-immunoreactive protein varied considerably in tissues from children with renal disease ranging from completely absent to strong and localized primarily to endothelial cells. However, there was no evidence that would link expression with a particular disease. Shown are high power views from a patient with IgA-nephropathy (Figure 3(a)), minimal change disease with focal segmental glomerulosclerosis (Figure 3(b)), and chronic transplant rejection (Figure 3(b)). Strong capillary expression of prostacyclin synthase in renal medulla was observed in the same biopsy (Figure 3(d)).

### 3.2. Prostacyclin Synthesis in Cultured Human Mesangial Cells

**Figure 4:**
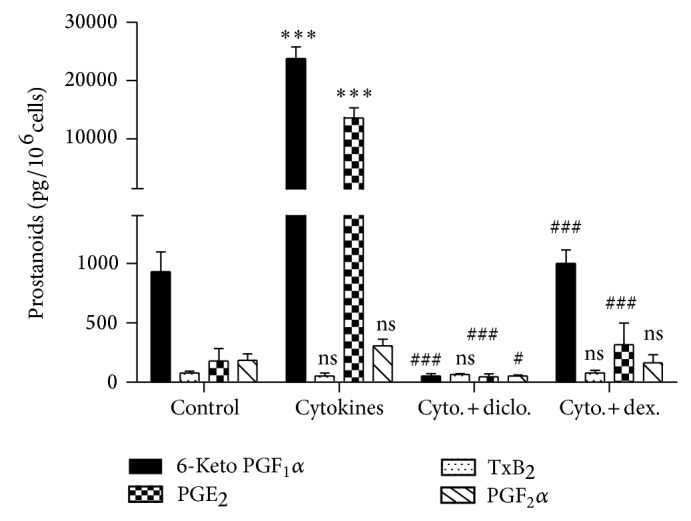
Pattern of COX1- and COX2-dependent prostanoid formation of human mesangial cells. HMCs were incubated for 20 h with cytokines in the absence or presence of diclofenac (1 *μ*M), dexamethasone (1 *μ*M), or vehicle. Prostanoid formation was measured in the supernatant. Data represent means (pg/10^6^ cells) ± SEM (*n* = 5). One-way analysis of variance Bonferroni's multiple comparison test was performed for each product. ^***^ *P* < 0.001 versus control; ^###^ *P* < 0.001 and ^#^ *P* < 0.05 versus cytokines; ns: nonsignificant.

*In Vitro Studies*. The profile of prostanoid synthesis in our cultured human mesangial cells (HMCs) is shown in Figure 4. Under basal conditions, mesangial cells produced predominantly PGI_2_ determined as 6-keto-PGF_1_ *α*, the primary product of PGI_2_ metabolic breakdown. Considerably smaller amounts of PGE_2_ and PGF_2_ *α* were observed and thromboxane (TX) type B_2_, the stable product of Tx type A_2_, was only barely detectable. Incubation of growth-arrested HMC from different donors with cytokines consisting of IL-1*β* (1 nM), TNF*α* (10 ng/mL), and IFN-*γ* (250 U/mL) resulted in increased expression of prostacyclin (24-fold) and PGE_2_ (65-fold). There was no apparent change in TxB_2_ and PGF_2_ *α* production. Coincubation of stimulated HMC with the nonsteroidal anti-inflammatory drug, diclofenac (1 *μ*M), resulted in a complete inhibition of prostanoid synthesis. In contrast, addition of the glucocorticoid dexamethasone (1 *μ*M) lowered cytokine-stimulated prostaglandin production to the same levels as seen under control conditions. The profile of exogenously added arachidonic acid metabolism is shown in Table 1. In a concentration-dependent manner, conversion to prostacyclin and PGE_2_ occurs. Once again, the levels of TxB_2_ and PGF_2_ *α*, prostanoids known to exert vasoconstrictive actions, were hardly detectable, even when the HMCs were challenged with arachidonic acid (20 *μ*M).

To gain further insight into the regulation of prostacyclin synthase activity following cytokine stimulation, the cyclooxygenase step was bypassed by the addition of exogenous PGH_2_, the immediate substrate for PGIS. As can be seen from the data in Table 2, compared to control, there was no significant difference in the conversion of PGH_2_ to 6-keto-PGF_1_ *α* in any of the cytokine or glucocorticoid treated samples.

**Figure 5:**
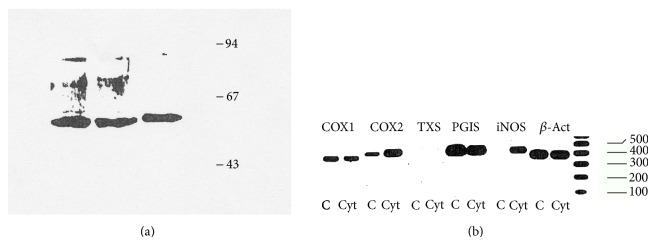
(a) Western blotting of prostacyclin synthase in HMC. Cell lysates (80 *μ*g) of cytokine treated HMC or control were stained with a polyclonal antibody for PGIS. Lane 1: control; lane 2: cytokine stimulation; lane 3: positive control (partially purified protein of bovine PGIS with a molecular mass of 52 KD); lane 4: molecular marker. (b) mRNA analysis of prostanoid generating enzymes and iNOS in HMC. RT-PCR analysis of the mRNA for COX1, COX2, TXS, PGIS, and iNOS from quiescent human mesangial cells (2 × 10^6^) was treated for 20 h with cytokines or vehicle. RNA preparation and PCR analysis were performed as described in Material and Methods. A 100 bp ladder is given on the right to evaluate the mass of the amplified fragments.

These data point towards a constitutive activity of prostacyclin synthase. To investigate this aspect at the protein level, Western blot analysis was performed using cell lysates of cytokine treated HMC or controls. The authenticity of the observed single band of approximately 52 kD was evidenced using the purified bovine enzyme as a positive control (Figure 5(a)).

Similar results, confirming the constitutive nature of the enzyme, were obtained addressing the corresponding mRNA expression (Figure 5(b)). Messenger RNA expression for both PGIS and COX1 was unaffected by cytokine stimulation, whereas expression of COX2 mRNA was markedly induced.

Interestingly, RT-PCR analysis failed to detect mRNA for TXS and suggested a negligible role for TxA_2_ as mesangial cell derived prostanoid. The extensive increase of inducible nitric oxide synthase mRNA under identical conditions was reported in HMC [19] and served as a control for cytokine activity.

## 4. Discussion

In the present study, it appear to be a strong expression of PGIS mRNA and immunoreactive protein in mesenchymal cells of developing human and mouse kidney, respectively. The greatest expression appeared to be subcortical, in the most immature part of the kidneys, namely, the nephrogenic cortex, in mesenchymal cells adjacent to ureteric buds. Previously, the regulation of PGIS expression during development has been shown during uterine development, with high levels of expression occurring in the most immature cells [3]. The strong expression of PGIS suggests that promoters of PGIS gene expression that have yet to be identified must be involved. Further studies, possibly including the investigation of miRNAs as potential regulators, are needed to identify the mechanisms leading to the upregulation of PGIS expression. Considering the multiple defects in renal development in PGIS-ko mice, including increased interstitial fibrosis [3], leads to speculation that prostacyclin plays an important role in the prevention of fibrosis and that this process may be modulated via PPAR-*β*/*δ* (given the normal renal phenotype in IP-ko-mice [9]).

Our study describes for the first time the intrarenal localization of prostacyclin synthase mRNA and immunoreactive protein in the healthy adult human kidney. Expression of PGIS mRNA and immunoreactive protein in vascular structures appears to confirm pharmacologic findings and is consistent with a role for prostacyclin as a vasodilator and inhibitor of platelet activation [20]. Expression of PGIS in the juxtaglomerular apparatus is in accordance with prostacyclin as mediator of renin release [21]. Expression of PGIS mRNA and immunoreactive protein was less prominent and discernible over mesangial fields. The exact cellular localization (mesangial or endothelial cells) could not be identified. Consistent with previous reports [22], there was no observable tubular expression of PGIS. This agrees with the findings of earlier studies which did not show a role of endogenous prostacyclin on tubular transport [20].

We investigated whether PGIS, like other developmentally regulated genes [23], is upregulated in glomerular disease. We observed variable degrees of PGIS expression in human kidney disease, though the expression was frequently higher than that seen in samples from healthy tissue controls. The interpretation of these observations was limited by the number of biopsies available for study and the multiple underlying diseases, precluding any correlation of PGIS expression with a particular glomerulopathy. The majority of cases showed upregulation of PGIS in glomerular and peritubular endothelial cells, which suggests a common inducer (Figures 3(a) and 3(b)). In some biopsies, however, inter- (Figure 3(c)) and intraglomerular (Figure 3(d)) expression was quite variable, possibly altered by local hemodynamics. Serial sections stained for COX1 and COX2 (data not shown) did not reveal coexpression with PGIS, arguing for differential modes of regulation (of COX1, COX2, and PGIS, resp.). As endothelial cells, for instance, are known to synthesize prostacyclin, absence of COX1 or COX2 costaining with PGIS may simply reflect high specificity and reduced sensitivity of our immunostaining procedure.

Our observations with cultured human mesangial cells appear to confirm that prostacyclin [24] is the predominant mesangial prostanoid both under basal conditions and following cytokine stimulation. The increase in PGI_2_ synthesis was mirrored by PGE_2_ synthesis [24]. Similarly to PGI_2_, PGE_2_ is a potent vasodilator, dependent on specific receptors located on target cells.

Despite using highly selective and sensitive GC/MS/MS, we could only detect trace amounts of the vasoconstricting prostanoids, TxA_2_ and PGF_2_ *α*, from HMS. In contrast to cytokine induced PGE_2_ and PGI_2_ formation, we did not observe changes in TxA_2_ and PGF_2_ synthesis. There is evidence that these prostanoids are predominately formed products of human glomerular epithelial cells (unpublished observations). Thus, we suggest that the previously reported [25] formation of TxA_2_ or PGF_2_ *α* from human mesangial cells is possibly a consequence of contamination of the previous authors' work with visceral epithelial cells. The predominance of the vasodilatory prostanoids PGE_2_ and PGI_2_ suggests that mesangial prostanoid formation exerts a tonic vasodilatory effect on the glomerular capillary network.

In a different set of experiments, we sought to better understand the mechanisms involved in cytokine induced PGI_2_ synthesis. Exposure of HMC to cytokines resulted in a 24-fold increase of PGI_2_ synthesis, but there was no evidence of PGIS mRNA and protein having been induced. In contrast, there was a marked induction of COX2 mRNA and protein. These results are in agreement with previous studies [26] and suggest that the limiting step in HMC-derived PGI_2_ synthesis is the regulation of COX2 expression. To substantiate the concept that COX2 levels regulate prostanoid synthesis in HMCs, we investigated the effect of dexamethasone on cytokine induced PGI_2_ prostaglandin formation. Dexamethasone is known to inhibit stimulated expression of both COX2 protein and mRNA through its action on transcriptional and posttranslational mechanisms. In cytokine-stimulated HMC, dexamethasone repressed COX2 mRNA and protein expression in parallel with a decrease in prostanoid formation to levels seen inside control. As dexamethasone failed to affect expression of the PGIS gene, our data appears to additionally support the concept that COX2 is the key step in controlling prostanoid formation in HMCs. Exposure of cytokine-stimulated HMCs to the nonsteroidal anti-inflammatory drug diclofenac, which inhibits both COX1 and COX2 activities, reduced prostanoid synthesis to levels below those seen in controls. These findings appear to suggest that COX1 contributes to basal HMC-derived prostanoid formation. Accordingly, we also observed constitutive expression of COX1 mRNA.

Our data appear to be in contrast with the observations made in bovine aortic endothelial cells, which has demonstrated the upregulation of prostacyclin mRNA following TNF*α* stimulation [11]. However, our findings are in line with those from experiments conducted in human venous umbilical endothelial cells [10]. Therefore, we propose that cell-specific regulation of prostacyclin synthase may occur under certain conditions or that the apparent difference in finding is a consequence of species differences. This notion is a supported pattern of organization of the human prostacyclin synthase gene, which was recently determined and uncovered consensus sequences for Sp-1, Ap-2, and, interestingly, NF-*κ*B in the promoter region [11]. NF-*κ*B is known as a transcriptional activator involved in the transmission of proinflammatory responses and could be a target for PGIS transcriptional regulation. The signalling pathway triggering PGIS induction in human systems therefore seems to be more complex and may involve a complex cytokine network (in a fashion similar to that recently proposed for nitric oxide synthase) or the presence of yet unknown mediators.

Nevertheless, our data appear to demonstrate clearly that regulation of PGI synthesis is regulated predominantly by COX2 activity, which serves to provide PGH to the constitutively expressed PGIS.

When considering our current findings in conjunction with what is already known about prostacyclin synthase, it appears that our data shows it to be under the influence of a developmentally regulated gene which is often reexpressed in glomerular disease. These* in vivo* findings appear to be in contrast with the constitutive expression of PGIS in primary human mesangial cells. Nevertheless, our data point to hitherto unknown regulators of prostacyclin synthase expression.

## Figures and Tables

**Table 1:** Prostanoid formation of cytokine-stimulated HMC from different concentrations of exogenously added arachidonic acid.

	6-Keto-PGF_1_ *α*	PGE_2_	TxB_2_	PGF_2_ *α*
AA (0 *μ*M)	242 ± 42^***^	78 ± 8^***^	96 ± 7^ns^	40 ± 13^ns^
AA (2 *μ*M)	6590 ± 300^***^	6120 ± 370^***^	110 ± 7^ns^	560 ± 6.6^ns^
AA (5 *μ*M)	11350 ± 250^***^	10390 ± 610^***^	83 ± 7^ns^	116 ± 7^ns^
AA (20 *μ*M)	14800 ± 100^***^	13500 ± 200^∗∗(a)^	100 ± 24^ns^	226 ± 40^∗∗(b);##(c)§(d)^

HMC were stimulated with cytokines for 20 h and later they were incubated with the depicted concentrations of exogenous arachidonic acid (AA) or only buffer (0 *μ*M) for 15 min at 20°C. Prostaglandins generated were extracted from the supernatant as described and analyzed by GC/MS/MS method. Values are depicted as means ± SEM (pg/10^6^ cells) of three experiments. One-way analysis of variance Bonferroni's multiple comparison test. ^***^ *P* < 0.001 for all combinations. ^(a)∗∗^ *P* < 0.01 for 5 *μ*M versus 20 *μ*M; ^(b)∗∗^ *P* < 0.01 for 0 *μ*M versus 20 *μ*M; ^(c)##^ *P* < 0.01 for 2 *μ*M versus 20 *μ*M, and ^(d)§^ *P* < 0.05 for 5 *μ*M versus 20 *μ*M; ns: not significant.

**Table 2:** Conversion of the precursor endoperoxide PGH_2_ to 6-Keto-PGF_1_ *α* in the presence of cytokines or dexamethasone.

	6-Keto- PGF_1_ *α* (ng/10^6^ cells)
Control	197 ± 14
Cytokines	139 ± 10^*^
Cytokines + dexamethasone (1 mM)	144 ± 7^*^

HMC were stimulated for 20 h with cytokines, cytokines plus dexamethasone, or vehicle. Following stimulation, the medium was aspirated and the cells were stimulated for 5 min at 20°C with 20 *μ*M PGH_2_ in phosphate buffered saline. The spontaneous decay of PGH_2_ in aqueous solution from a control experiment was considered. The figure consists of the mean of three experiments ± SEM. One-way analysis of variance Bonferroni's multiple comparison test: ^*^ *P* < 0.05 versus control.

## Abbreviations

PGIS: Prostacyclin synthase
TXS: Thromboxane synthase
COX: Cyclooxygenase
HMC: Human mesangial cell
GC/MS/MS: Gas chromatography/mass spectrometry
PPAR-*β*/*δ*: Peroxisome proliferator-activated receptor *β*/*δ*
IgA-GN: IgA-nephropathy
FSGS: Focal segmental glomerulosclerosis
PGI_2_: Prostaglandin I_2_
PGH_2_: Prostaglandin H_2_
TNT_*x*_: Tumour necrosis factor type alpha
PCR: Polymerase chain reaction
IL: Interleukin
Rh: Recombinant human.

## References

[1] Wu K. K., Liou J.-Y. (2005). Cellular and molecular biology of prostacyclin synthase. *Biochemical and Biophysical Research Communications*.

[2] Yokoyama C., Yabuki T., Inoue H. (1996). Human gene encoding prostacyclin synthase (PTGIS): genomic organization, chromosomal localization, and promoter activity. *Genomics*.

[3] Lim H., Gupta R. A., Ma W.-G. (1999). Cyclo-oxygenase-2-derived prostacyclin mediates embryo implantation in the mouse via PPAR*δ*. *Genes and Development*.

[4] Nasrallah R., Hébert R. L. (2005). Prostacyclin signaling in the kidney: implications for health and disease. *The American Journal of Physiology: Renal Physiology*.

[5] Hertz R., Berman I., Keppler D., Bar-Tana J. (1996). Activation of gene transcription by prostacyclin analogues is mediated by the peroxisome-proliferators-activated receptor (PPAR). *European Journal of Biochemistry*.

[6] Gupta R. A., Tan J., Krause W. F. (2000). Prostacyclin-mediated activation of peroxisome proliferator-activated receptor *δ* in colorectal cancer. *Proceedings of the National Academy of Sciences of the United States of America*.

[7] Fauti T., Müller-Brüsselbach S., Kreutzer M. (2006). Induction of PPAR*β* and prostacyclin (PGI2) synthesis by Raf signaling: failure of PGI2 to activate PPAR*β*. *FEBS Journal*.

[8] Yokoyama C., Yabuki T., Shimonishi M. (2002). Prostacyclin-deficient mice develop ischemic renal disorders, including nephrosclerosis and renal infarction. *Circulation*.

[9] Murata T., Ushikubi F., Matsuoka T. (1997). Altered pain perception and inflammatory response in mice lacking prostacyclin receptor. *Nature*.

[10] Spisni E., Bartolini G., Orlandi M., Belletti B., Santi S., Tomasi V. (1995). Prostacyclin (PGI2) synthase is a constitutively expressed enzyme in human endothelial cells. *Experimental Cell Research*.

[11] Hara S., Miyata A., Yokoyama C. (1994). Isolation and molecular cloning of prostacyclin synthase from bovine endothelial cells. *Journal of Biological Chemistry*.

[12] Weksler B. B. (1990). Heparin and acidic fibroblast growth factor interact to decrease prostacyclin synthesis in human endothelial cells by affecting both prostaglandin H synthase and prostacyclin synthase. *Journal of Cellular Physiology*.

[13] Krämer B. K., Kammerl M. C., Kömhoff M. (2004). Renal cyclooxygenase-2 (Cox-2). Physiological, pathophysiological, and clinical implications. *Kidney & Blood Pressure Research*.

[14] Kömhoff M., Gröne H.-J., Klein T., Seyberth H. W., Nüsing R. M. (1997). Localization of cyclooxygenase-1 and -2 in adult and fetal human kidney: implication for renal function. *The American Journal of Physiology: Renal Physiology*.

[15] Kömhoff M., Guan Y., Shappell H. W. (2000). Enhanced expression of cyclooxygenase-2 in high grade human transitional cell bladder carcinomas. *The American Journal of Pathology*.

[16] Siegle I., Klein T., Zou M.-H., Fritz P., Kömhoff M. (2000). Distribution and cellular localization of prostacyclin synthase in human brain. *Journal of Histochemistry and Cytochemistry*.

[17] Siegle I., Nüsing R., Brugger R., Sprenger R., Zecher R., Ullrich V. (1994). Characterization of monoclonal antibodies generated against bovine and porcine prostacyclin synthase and quantitation of bovine prostacyclin synthase. *FEBS Letters*.

[18] Schweer H., Watzer B., Seyberth H. W. (1994). Determination of seven prostanoids in 1 ml of urine by gas chromatography-negative ion chemical ionization triple stage quadrupole mass spectrometry. *Journal of Chromatography B: Biomedical Applications*.

[19] Nicolson A. G., Haites N. E., McKay N. G., Wilson H. M., MacLeod A. M., Benjamin N. (1993). Induction of nitric oxide synthase in human mesangial cells. *Biochemical and Biophysical Research Communications*.

[20] Hao C.-M., Breyer M. D. (2008). Physiological regulation of prostaglandins in the kidney. *Annual Review of Physiology*.

[21] Fujino T., Nakagawa N., Yuhki K.-I. (2004). Decreased susceptibility to renovascular hypertension in mice lacking the prostaglandin I_2_ receptor IP. *The Journal of Clinical Investigation*.

[22] Vitzthum H., Abt I., Einhellig S., Kurtz A. (2002). Gene expression of prostanoid forming enzymes along the rat nephron. *Kidney International*.

[23] Lappin D. W. P., McMahon R., Murphy M., Brady H. R. (2002). Gremlin: an example of the re-emergence of developmental programmes in diabetic nephropathy. *Nephrology, Dialysis, Transplantation*.

[24] Ardaillou N., Nivez M. P., Striker G., Ardaillou R. (1983). Prostaglandin synthesis by human glomerular cells in culture. *Prostaglandins*.

[25] Floege J., Topley N., Wessel K. (1990). Monokines and platelet-derived growth factor modulate prostanoid production in growth arrested, human mesangial cells. *Kidney International*.

[26] Hao C.-M., Breyer M. D. (2007). Roles of lipid mediators in kidney injury. *Seminars in Nephrology*.

